# Neurotoxicity Assessment of Amicarbazone Using Larval Zebrafish

**DOI:** 10.3390/toxics12110783

**Published:** 2024-10-28

**Authors:** Seung-Hwa Baek, Yeonhwa Kim, Suhyun Kim, Hae-Chul Park

**Affiliations:** 1 Medical Science Research Center, Korea University Ansan Hospital, Ansan 15355, Gyeonggi-do, Republic of Korea; micro340@gmail.com; 2 Zebrafish Translational Medical Research Center, Korea University, Ansan 15355, Gyeonggi-do, Republic of Korea; yeonhwa0215@gmail.com; 3 Department of Biomedical Sciences, College of Medicine, Korea University, Seoul 04763, Republic of Korea

**Keywords:** zebrafish, amicarbazone, central nervous system, neurotoxicity, aquatic toxicology

## Abstract

Amicarbazone (AMZ), a triazolinone herbicide widely applied in agriculture, is known to inhibit photosystem II in target plants, disrupting photosynthesis and causing oxidative stress that leads to weed mortality. Despite its widespread use, the developmental and neurotoxic effects of AMZ on aquatic organisms remain underexplored. This study assesses the impact of AMZ exposure on zebrafish (*Danio rerio*) embryos/larvae, focusing on developmental toxicity and neurotoxicity. Zebrafish were exposed to AMZ at various concentrations to evaluate survival, malformations, heart rate, and behavior. Significant developmental defects, including reduced survival rates, increased malformations, and decreased heart rates, were observed in zebrafish embryos exposed to AMZ, particularly at higher concentrations. Additionally, behavioral assays revealed decreased locomotor activity, particularly at concentrations of 100 and 200 mg/L. Moreover, AMZ exposure disrupted motor axon formation, oligodendrocyte development, and the expression of key genes involved in neurodevelopment. The downregulation of cholinergic, dopaminergic, and serotonergic signaling pathways was also identified, indicating neurotoxicity. These findings highlight AMZ’s potential to induce both developmental and neurotoxic effects in zebrafish and suggest the need for further research on its long-term ecological impacts.

## 1. Introduction

Amicarbazone, a triazolinone herbicide, is widely used in agriculture to control various weed species, particularly in crops like sugarcane, corn, and turfgrass [1]. It was first registered by the US Environmental Protection Agency (EPA) for use on corn in 2005 and is commonly applied on farms and golf courses in the US and Australia. AMZ disrupts the photosystem II (PSII) complex in target plants, inhibiting photosynthesis by disrupting the electron transport chain, which generates reactive oxygen species and ultimately causes cell death in susceptible weeds [2].

The chemical properties of AMZ reveal its high solubility in polar organic solvents (e.g., acetone and dimethyl sulfoxide > 250 g/L) but limited solubility in water (4.6 g/L at 20 °C, pH 4.9) [2]. It is relatively stable under environmental conditions, with a photolytic half-life of 66 days in natural water at pH 7 and a hydrolysis half-life of 64 days at pH 7. Furthermore, AMZ is moderately persistent in aerobic soils, with a field half-life ranging from 28 to 44 days and a laboratory half-life of approximately 50 days [3]. These attributes enable AMZ to degrade slowly and potentially contaminate aquatic ecosystems via spray drift or runoff. The EPA has reported the detection of AMZ in both groundwater and surface water, with peak concentrations reaching 136 µg/L in groundwater and 33.3 µg/L in surface water (96 h average) [4]. Such contamination poses a risk to aquatic ecosystems, as prolonged exposure to AMZ can negatively impact the growth and reproduction of aquatic organisms [5].

Although AMZ exhibits relatively low acute toxicity in mammals [2], its toxic effects on aquatic organisms are variable. For instance, AMZ is highly toxic to algae (EC50 of 0.035 mg/L in marine diatoms) and higher aquatic plants (EC50 of 0.21 mg/L) [3]. Invertebrates such as Daphnia magna exhibit moderate sensitivity (EC50 of 0.21 mg/L), while fish, including rainbow trout and bluegill sunfish, demonstrate a much lower acute toxicity, with LC50 values exceeding 120.4 mg/L and 118 mg/L, respectively [6].

Neurotoxic effects of AMZ have been observed in mammalian models, with studies in rats indicating dose- and time-dependent reductions in motor activity, while mice exposed to 100 mg/kg of AMZ displayed motor impairment and increased nociception [4]. However, studies on the developmental neurotoxicity of AMZ in aquatic organisms are lacking.

Zebrafish (*Danio rerio*), a widely used model organism in toxicological research, provides a valuable system for studying the developmental and neurotoxic effects of chemicals. Zebrafish share significant neurodevelopmental similarities with mammals and offer advantages in bioimaging and behavioral assessments [7,8,9,10]. Despite AMZ’s widespread use as an herbicide, there is limited information regarding its toxicity in zebrafish. This study aims to address this gap by characterizing the developmental and neurotoxic effects of AMZ on zebrafish embryos and larvae, focusing on behavioral changes, motor neuron development, oligodendrocyte formation, and gene expression related to neural development.

## 2. Materials and Methods

### 2.1. Materials

Amicarbazone (AMZ; CAS 129909-90-6; >98% purity), dimethyl sulfoxide (DMSO; CAS 67–68-5; ≥99.9% purity), 3,4-dichloroaniline (3,4-DCA, CAS 95-76-1; ≥97.5% purity), and tricaine (MS-222; CAS 886-86-2; 97.5-102.5% purity) were obtained from Sigma-Aldrich (St. Louis, MO, USA). AMZ was dissolved in DMSO at 400 g/L and used as a stock solution.

### 2.2. Zebrafish Lines and Developmental Toxicity Test

Wild-type, Tg(MBP:EGFP) zebrafish expressing EGFP in oligodendrocytes [11] and Tg(Olig2:dsRed) [12] zebrafish expressing dsRed in motor neurons and axons were utilized in this study. The zebrafish were housed in an automated aquaculture system at 28.5 °C with a 14:10 h light:dark cycle. Zebrafish embryos were staged based on hours post fertilization (hpf). Wild-type zebrafish cultures and embryo exposures were performed as previously described [13]. For the developmental toxicity test, zebrafish embryo collection and chemical treatment followed OECD guideline No. 236. Briefly, 50 normally developing embryos at 4 hpf were exposed to 200 μL AMZ at concentrations of 100, 200, 300, 400, and 800 mg/L in a 96-well plate (SPL, Pocheon, Republic of Korea), with one embryo per well. Control groups were treated with 0.1% DMSO, including a negative control (E3 medium; 15 mM NaCl, 0.5 mM KCl, 1 mM CaCl_2_, 1 mM MgSO_4_, 0.15 mM KH_2_PO_4_, 0.05 mM NH_2_PO_4_, and 0.7 mM NaHCO_3_) and a positive control (3,4-DCA, 4 mg/L, OECD guidelines No. 236). AMZ solutions were replaced daily at the same concentration until 120 hpf. Three repeated experiments were conducted independently. Embryos and larvae were monitored daily for survival, hatching, and malformations at 24, 48, 72, and 120 hpf. At 120 hpf, larvae were immobilized in 1.5% low-melting-point agarose, and their heart rates were measured for 30 s using a stereomicroscope (Leica, Tokyo, Japan). Mortality was determined by the absence of heartbeats according to the OECD guidelines (No. 236). Anomalies included cardiac malformations, pericardial edema, uninflated swim bladders, and yolk sac edema. Five morphological images were captured from randomly selected embryos in each group using a microscope. As malformations and reduced heart rates were evident from 300 mg/L at 120 hpf, experimental concentrations of 100 mg/L and 200 mg/L were chosen to test for neurotoxicity.

### 2.3. Transgenic Zebrafish Imaging

Tg(Olig2:dsRed) zebrafish embryos were exposed from 4 hpf to 72 hpf at AMZ concentrations of 100 and 200 mg/L with 0.1% DMSO in 0.003% (*w*/*v*) PTU solution for up to 72 hpf to assess motor axon development without affecting the myelin sheath. The motor axon length was measured using the conventional method for assessing motor axonal degeneration [14,15,16]. Briefly, motor axon length was quantified from the beginning of the axon emerging from the cell body to the end of the muscle. The number of primary branches was counted based on lateral branches, called axonal collaterals, extending directly from the axon. Tg(MBP:EGFP) zebrafish embryos were similarly exposed to the same AMZ concentrations and 0.1% DMSO in 0.003% (*w*/*v*) PTU solution for up to 120 hpf. The number of oligodendrocytes was quantified as previously described [17]. A total of 20–30 embryos per well were exposed to each concentration in a 6-well plate (SPL, cat. no. 30006). Embryos expressing dsRed and EGFP were selected using a fluorescence microscope (SMZ18; Nikon, Tokyo, Japan). For motor axon and oligodendrocyte imaging, live transgenic zebrafish larvae were anesthetized with an anesthetic (MS-222, 0.03%), positioned laterally in groups of 5–6 in a spheroid dish (No.110350, SPL), and embedded in 1.5% low-melting-point agarose. All fluorescent images were captured in the 5-somite region above the yolk extension with a spinning confocal microscope (CSU-X1, Nikon, Tokyo, Japan). Each experiment was independently performed in triplicate, with five to ten embryos imaged at a time, and the results of the three replicates were combined for statistical analysis. Motor axon length and branching and oligodendrocyte counts were measured using NIS-Elements AR Analysis 4.30 software (Nikon, Tokyo, Japan).

### 2.4. Behavior Analysis

Behavior analysis was conducted on wild-type zebrafish larvae exposed to 0, 100 and 200 mg/L AMZ at 120 hpf. Individual larvae were placed in 48-well plates (*n* = 48 per group) with fresh E3 medium (0.5 mL) and observed. Each experiment was performed in triplicate. After a 2 h acclimation period, the light/dark behavior test commenced, beginning with a 20 min light phase, followed by two alternating cycles of 5 min dark and 5 min light state, ending with a final 20 min of the dark phase. The test was conducted using the DanioVision Observation Chamber (Noldus, Wageningen, The Netherlands) according to a previously published protocol [17]. To analyze the recorded video, EthoVision XT software (Ver 16.0.1536, Noldus) was used to quantify locomotor activity based on average velocity (mm/s).

### 2.5. RT-qPCR Analysis

After the behavioral experiment was completed, all zebrafish larvae were retrieved, and RT-qPCR experiments were performed. Following the manufacturer’s protocol, total RNA was extracted from pools of 48 larvae at 120 hpf with TRIzol reagent (Life Technologies (Thermo Scientific, Rockford, IL, USA). cDNA was synthesized using AccuPower RT PreMix (BIONEER Corporation, Daejeon, Republic of Korea). The quality of the total RNA samples was evaluated by measuring the OD260/OD280 ratio. Real-time qPCR analysis was conducted using SsoFast™ EvaGreen^®^ Supermix (Biorad Corporation, Hercules, CA, USA) and an ABI 7500 Sequence Detection System (Applied Biosciences, Foster City, CA, USA). The primer sequences are listed in Table 1. PCR amplifications of the samples were carried out using 40 cycles of 5 s at 95 °C and 20 s at 60 °C. After RT-qPCR, a melting curve analysis confirmed PCR product specificity. All reactions were performed in triplicate, and relative expression levels normalized to *beta-actin* [18] were determined using the 2^−ΔΔDCT^ method [19].

### 2.6. Statistical Analysis

Statistical analysis and graph generation were conducted using GraphPad Prism 9 (GraphPad Software, Inc., La Jolla, CA, USA). Two-way analysis of variance (ANOVA) was performed to analyze the survival rate depending on time, locomotor responses, and RT-qPTR data among experimental groups exposed to AMZ, followed by Bonferroni’s multiple comparisons test for post hoc analysis. One-way ANOVA with Tukey’s multiple comparison method was used to assess developmental toxicity, the length of the motor axon, and the number of oligodendrocytes. All data are expressed as means ± standard deviation (SD). A *p*-value < 0.05 indicates statistical significance.

## 3. Results

### 3.1. Developmental Parameters

**Figure 1:**
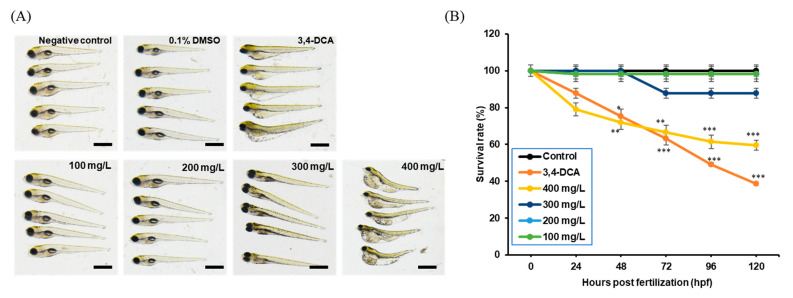
Toxic effects of AMZ on the morphology and mortality activity of zebrafish larvae. (**A**) The embryo phenotypes at 120 hpf in the unexposed and AMZ-exposed groups. (**B**) The survival rate of zebrafish at 120 hpf after exposure to AMZ. Significant differences from the control are indicated by * *p* < 0.05, ** *p* < 0.01, and *** *p* < 0.001. Data are expressed as the mean ± SD from 3 replicates (*n* = 50 embryos per group). Scale bar, 100 μm.

Morphological assessments of zebrafish embryos exposed to AMZ revealed significant developmental disruptions (Table 2). At 120 h post fertilization (hpf), exposure to 400 mg/L of AMZ resulted in a 40.3% reduction in the survival rate and a 7.7% decrease in the hatching rate. Malformations were prominent in the 300 mg/L (56%) and 400 mg/L (94%) groups, with specific abnormalities such as yolk and pericardial edema, spinal and tail curvature, and an absence of swim-bladder formation in the 300 mg/L group (Figure 1). Additionally, heart rate was markedly reduced by 44% at 300 mg/L (*p* < 0.001) and 76% at 400 mg/L (*p* < 0.001) in AMZ-exposed embryos.

### 3.2. Locomotor Behavior of Zebrafish Larvae

**Figure 2:**
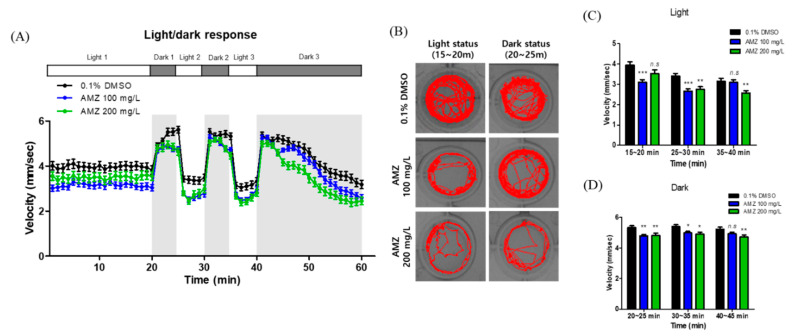
Locomotor behavior test in AMZ-exposed zebrafish larvae. Locomotor behavior was analyzed in zebrafish larvae exposed to 0, 100, and 200 mg/L AMZ at 120 hpf. (**A**) Zebrafish larvae were acclimated to light for 20 min, followed by two alternating cycles of 5 min of light and dark phases. (**B**) Images captured during the 5 min transitions between light and dark status. (**C**,**D**) The velocity of the larvae was recorded during light and dark status in the photoperiod stimulation test. Data are presented as the mean ± SD from five replicates (*n* = 48 larvae per group). *n.s*—not significant. * *p* < 0.05, ** *p* < 0.01, and *** *p* < 0.001 compared to the control group (0.1% DMSO).

The locomotor activity of zebrafish larvae exposed to AMZ was monitored under alternating light–dark conditions. Zebrafish exposed to 100 and 200 mg/L of AMZ exhibited significantly reduced velocity compared to controls, with diminished locomotor responses across light and dark cycles (Figure 2).

### 3.3. Motor Axonopathy

**Figure 3:**
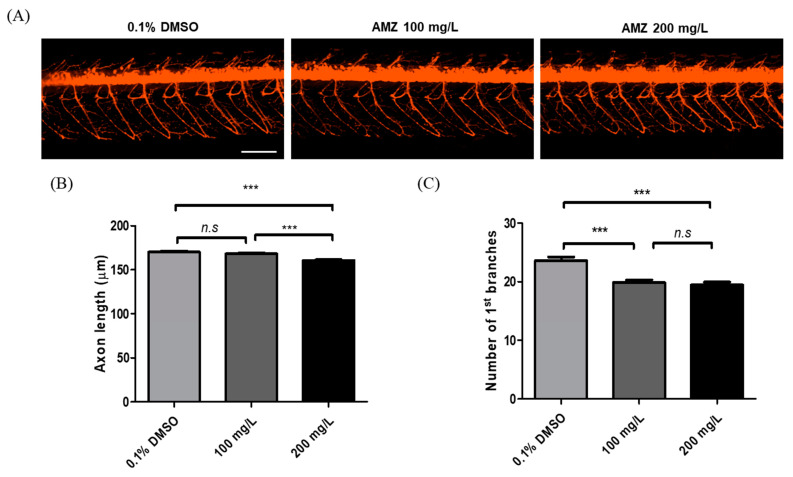
AMZ exposure affected axonal growth in zebrafish larvae. (**A**) Red fluorescence images of axon length in Tg (Olig2:dsRed) zebrafish at 72 hpf. (**B**) Quantitative measurements of axon length. (**C**) Number of 1st branches of axonal length. Data are expressed as the mean ± SD (60 axons from 15 embryos per group). *n.s*—not significant. *** *p* < 0.001 compared to the control (0.1% DMSO). Scale bar, 100 μm.

Next, we assessed the effect of AMZ exposure on motor axon formation. Motor axonal length measured from the sagittal plane provided an accurate representation of the 3D axonal trajectory along the Z axis (Figure 3A). AMZ exposure at 200 mg/L significantly reduced motor axon length and decreased the number of primary branches compared to controls, with no significant changes observed at 100 mg/L (*p* < 0.001, Figure 3).

### 3.4. Myelination

**Figure 4:**
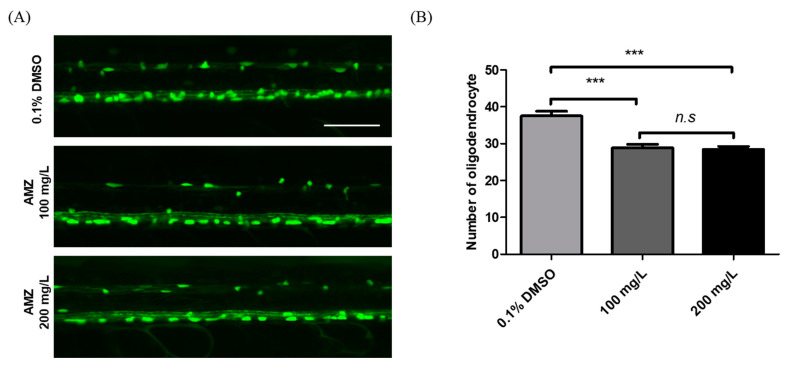
Toxicological effects of AMZ on oligodendrocytes of zebrafish larvae at 5 dpf. (**A**) Lateral views of the spinal cord of Tg(MBP:EGFP) at 120 hpf. The oligodendrocytes expressed EGFP fluorescence. (**B**) The graph compares the number of oligodendrocytes per three somites in zebrafish treated with 0.1% DMSO and 100 and 200 mg/L AMZ (*n* = 26 larvae for DMSO control, *n* = 24 larvae AMZ 100 mg/L, and *n* = 30 larvae for AMZ 200 mg/L). All data are expressed as the mean ± SD. *n.s*—not significant. *** *p* < 0.001 compared to the control (0.1% DMSO). Scale bar, 50 μm.

Myelination is critical for the development, function, and motor activity of the nervous system. To assess the impact of AMZ on myelination, we examined the formation of oligodendrocytes, which produces myelin sheaths in the central nervous system (CNS) (Figure 4A). Zebrafish larvae exposed to AMZ exhibited reductions in oligodendrocyte numbers (*p* < 0.001, Figure 4), indicating a disrupted myelination processes (*p* < 0.001; Figure 4B).

### 3.5. Changes in Gene Expression

**Figure 5:**
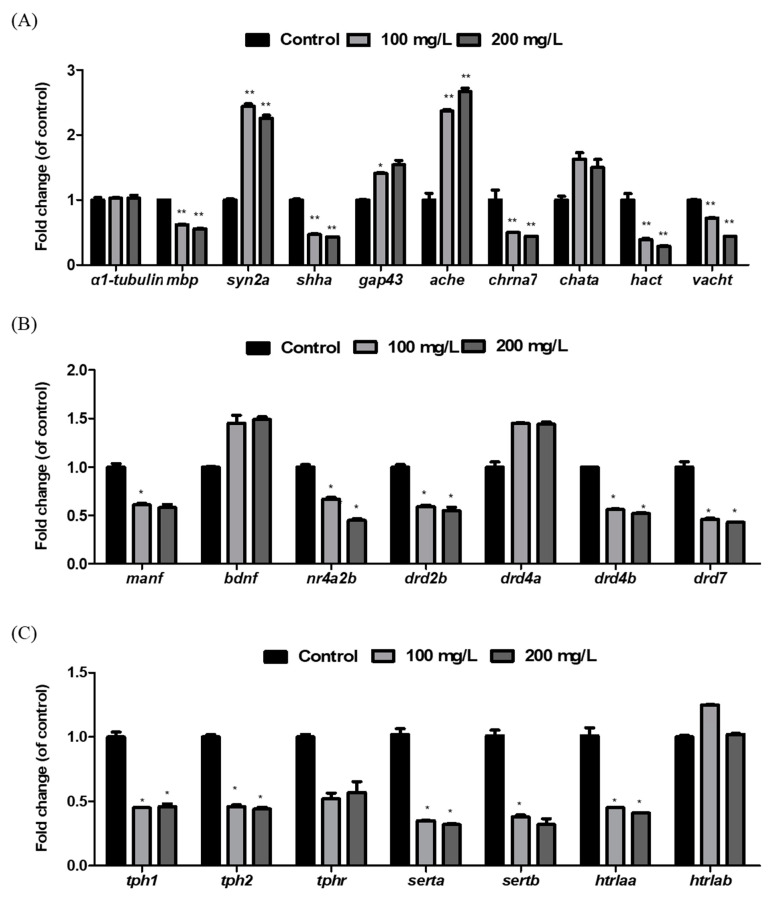
Transcription of specific development and neurotransmitter system-related genes in AMZ-exposed zebrafish larvae. (**A**) CNS development (*a1-tubulin*, *mbp*, *syn2a*, *shha*, and *gap43*) and the cholinergic system (*ache*, *chrna7*, *chata*, *hact*, and *vacht*). (**B**) Dopaminergic system (*manf*, *bdnf*, *nr4a2b*, *drd2b*, *drd4a*, *drd4b*, and *drd7*). (**C**) Serotonergic system (*tph1*, *tph2*, *tphr*, *serta*, *sertb*, *htr1aa*, and *htr1ab*). All data are expressed as the mean ± SD value from three replicates (48 larvae per replicate). * *p* < 0.05, and ** *p* < 0.01 compared to the control (0.1% DMSO).

The mRNA expression levels of genes involved in nervous system development were evaluated in zebrafish larvae exposed to AMZ at 120 hpf. Gene expression analysis revealed significant downregulation of key genes involved in neural development, including *α1-tubulin*, *myelin basic protein (mbp)*, *synapsin 2a (syn2a)*, *sonic hedgehog a (shha)*, and *growth-associated protein 43 (gap43)* in AMZ-exposed larvae (Figure 5A). At 100 mg/L AMZ, genes were downregulated by 1.6 and 2.1 fold, while at 200 mg/L, the downregulation increased to 1.8 and 2.3 fold. Similarly, presynaptic cholinergic genes, such as *nicotinic acetylcholine receptor subunit 7 (chrna7)*, *high-affinity choline transporter (hact)*, and *vesicular acetylcholine transporter (vacht)*, were significantly downregulated (2.3, 3.5, and 2.3 fold, respectively) at 200 mg/L AMZ (*p* < 0.05). Transcription of *acetylcholinesterase (ache)* was notably upregulated, increasing by 2.4 fold at 100 mg/L and by 2.7 fold at 200 mg/L AMZ. Dopaminergic system-related genes, such as *mesencephalic astrocyte-derived neurotrophic factor (manf); brain-derived neurotrophic factor (bdnf); nuclear receptor subfamily 4*, *group A*, *member 2b (nr4a2b)*; and dopamine receptors (*drd2b*, *drd4a*, *drd4b*, and *drd7*), showed significant downregulation. At 200 mg/L AMZ, *manf* and *nr4a2b* were downregulated by 1.7 and 2.2 fold, respectively (Figure 5B). Downregulation of dopamine receptors *drd2b*, *drd4b*, and *drd7* was observed at 1.8, 1.9, and 2.3 fold, respectively, following exposure to 200 mg/L AMZ. The expression levels of genes associated with the serotonergic system, such as tryptophan hydroxylase (*tph1*, *tph2*, and *tphr*), serotonin transporters (*serta* and *sertb*), and 5-hydroxytryptamine receptors (*htr1aa* and *htr1ab*) were also evaluated. The expression levels of *serta*, *sertb*, and *htr1aa* were found to be downregulated by 3.1, 3.1, and 2.4-fold, respectively, in larvae exposed to 200 mg/L AMZ (*p* < 0.05) (Figure 5C).

## 4. Discussion

AMZ, a selective herbicide used to control annual broadleaf weeds in corn crops [1], induced developmental toxicity in zebrafish larvae exposed from 4 to 120 hpf at concentrations of 300 and 400 mg/L. Observed effects included decreased survival and heart rates, as well as increased malformation rates (Table 2, Figure 1). A recent study also demonstrated that exposure to high concentrations of sulfentrazone, another triazolinone member; delayed yolk sac absorption; and disrupted hatching and altered heart rates in zebrafish embryonic stages at 96 hpf [20]. This suggests that triazolinone herbicides such as AMZ can induce developmental toxicity.

The assessment of locomotor behavior in zebrafish larvae serves as a critical indicator of neurodevelopment [17,21,22,23,24]. AMZ exposure resulted in decreased locomotor activity associated with neurotoxicity. Comparable effects have been observed with other herbicides, such as haloxyfop-p-methyl, which notably diminished swimming speed and altered movement dynamics in larvae [25]. Additionally, exposure to tiafenacil, a novel protoporphyrinogen oxidase (PPO)-inhibiting herbicide, reduced locomotor activity [26].

Recent findings also revealed that bixafen, a methylpyrazole carboxamide fungicide, induces neurotoxicity by impairing motor neuron axon growth and axonal branching in zebrafish larvae, significantly reducing locomotor activity [27]. Exposure to titanium dioxide nanoparticles (nano-TiO2) similarly affects zebrafish larval development, causing neurobehavioral alterations and inhibition of motoneuron axonal growth [28]. Axonal branching is a dynamic process involving the continuous addition and withdrawal of axonal branches influenced by neural activity [29]. The chronic blockade of neuronal activity with various drugs, such as tetrodotoxin (TTX), significantly reduces branch additions in specific layers, emphasizing the interplay between laminar specificity and activity-dependent mechanisms of thalamocortical axonal branching [30,31]. Tris (2-butoxyethyl) phosphate (TBOEP) exposure was also found to impact spontaneous movement, locomotor patterns, and swimming speed in developing zebrafish larvae, significantly inhibiting secondary motoneuron axonal growth at concentrations of 1500 and 2500 μg/L TBOEP [32]. These studies emphasize that exposure to widely used chemicals during developmental stages can result in neurotoxicity, affecting motor neurons and behavioral changes.

We confirmed the altered expression of CNS-related genes, including *α1-tubulin*, *mbp*, *syn2a*, *shha*, and *gap43*. *α1-tubulin* encodes an intermediate filament protein essential for microtubule cytoskeleton development or axon and dendrite regeneration, which is crucial for the formation of the nervous system [33]. *mbp* plays a role in regulating myelin levels in CNS axons, which are integral to myelination [34], while *syn2a* contributes to synapse formation [35]. *shha* serves as a signaling molecule in the nervous system [36], and *gap43* is involved in neuronal repair [37]. These genes are considered indicators of neurodevelopmental processes and have been associated with neurobehavioral changes in zebrafish [38]. AMZ exposure led to the downregulation of *mbp* and *shha* gene expression. Specifically, the reduction in mbp gene expression corresponded with decreased oligodendrocyte formation, thereby impairing myelination (Figure 4). These results validate the effect of AMZ in reducing myelination and affecting nervous system signaling molecules.

Presynaptic cholinergic genes *ache*, *chata*, *hact*, and *vacht* are expressed in the motoneurons [39]. In this study, the transcription of presynaptic cholinergic transcripts *chrna7*, *hact*, and *vacht* was significantly suppressed after exposure to AMZ. The cholinergic system, a pivotal branch of the autonomic nervous system, is involved in memory, blood pressure, digestion, movement, heart rate regulation, and various other functions [40]. The *chrna7* gene encodes the alpha-7 nicotinic acetylcholine receptor, which is crucial for cholinergic signaling in the brain, responding to acetylcholine binding [41]. Acetylcholine synthesis, storage, and release requires the expression of *vacht* and *chata* [42], with *vacht* facilitating acetylcholine secretion from neuronal secretory organelles [43] and *chata* catalyzing its biosynthesis in cholinergic neurons [44]. Acetylcholine is subsequently transported to the presynaptic neurons by *hact* [39]. Therefore, it is suspected that AMZ inhibits acetylcholine synthesis, thereby affecting neurotransmitter transmission to neurons, and simultaneously affects acetylcholine binding, which could explain the decrease in movement. Additionally, elevated *ache* mRNA, as demonstrated in this study, can increases acetylcholinesterase (AChE) enzymes activity, leading to excessive acetylcholine hydrolysis, exacerbating negative behavioral responses in zebrafish larvae. Our results also confirm that the significant increase in *ache* gene activity due to AMZ exposure likely contributes to reduced motility. Next, genes linked to dopamine, such as *manf*, *nr4a2b*, and *bdnf*, were also examined. In vertebrates, dopamine plays a central role in cognition, sociability, motor function, and immune system regulation via various receptors [45]. *manf* is essential for maintaining and regulating dopamine levels during dopaminergic neuron development [46,47], while *nr4a2b* is responsible for dopaminergic neuron differentiation [48]. *bdnf* regulates neuronal growth, differentiation, and repair [49]. Dopamine receptors, which are G protein-coupled, exist in at least five subtypes, namely D1 (*drd1* and *drd7*), D2 (*drd2a* and *drd2b*), D3 (*drd3*), D4 (*drd4a*, *drd4b*, and *drd4c*), and D5 (*drd5*) [50]. Previous studies hypothesized that *drd2* downregulation is associated with decreased zebrafish larval motility through dopamine signaling suppression by insecticides [51]. Moreover, *drd2a* and *drd2b* gene expression correlates with zebrafish boldness, with higher expression levels in bold male zebrafish, confirming the involvement of dopamine D2 receptors in locomotor behavior [52,53]. Our study showed significant downregulation of *drd2b* and *drd4b*, which are crucial genes in dopamine signaling, following AMZ exposure. This downregulation likely contributed to reduced dopamine content, potentially explaining the decreased behavioral activity observed in zebrafish larvae.

Serotonin, a neurotransmitter, serves as a key behavioral regulator in both vertebrates and invertebrates. Deficiencies in the serotonin system are associated with various behavioral disorders and pathological functional changes in the central nervous system [54,55,56]. The synthesis of serotonin is mediated by tryptophan hydroxylases (*tph1*, *tph2*, and *tphr*), which are the rate-limiting enzymes in this process [57]. Serotonin transporters (*serta* and *sertb*) and serotonin receptors (*htr1aa* and *htr1ab*) are essential for synaptic serotonin neurotransmission [58]. The serotonin transporter functions as both a symporter and antiporter within the presynaptic membrane of the CNS, facilitating the removal and resorption of serotonin [59]. Xianfeng et al. [60] discovered that exposure to PBDEs damaged neural development in zebrafish larvae by impairing serotonin neurotransmission, which is attributed to the genetic inhibition of serotonin receptors (*htr1aa* and *htr1ab*) and serotonin transporters (*serta* and *sertb*). The reduction in *tph1*/*tph2* expression level, likely resulting from AMZ’s damaging effects, may induce behavioral changes by modulating serotonin. The reduced transcript levels of *htr1aa*/*htr1ab* and *serta*/*sertb* suggest that AMZ could impair neurodevelopment in zebrafish larvae through its impact on serotonergic neurotransmission.

According to data from the US Geological Survey (USGS) and the EPA, numerous widely used pesticides have been detected in both surface and groundwater sources [61]. The risk of elevated and prolonged exposure remains a concern, as studies have demonstrated that heightened herbicide and pesticide concentrations in surface water, particularly from April to July, correlate with an increased incidence of birth defects in newborns [62]. Although the concentrations tested in this study exceeded the predicted environmental residues of AMZ in surface and groundwater, the experimental concentrations were below sublethal levels (LC20, 310 mg/L; LC50, 460 mg/L; Figure S1) Therefore, while this study has limitations in that the collected data do not directly reflect environmental exposure levels, it provides foundational data on the toxic effects of high concentrations of AMZ.

## 5. Conclusions

We found that AMZ exposure not only affected development but also caused neurotoxicity, including behavioral disturbances in zebrafish. In addition, AMZ exposure could cause significant negative changes in the transcription of several genes involved in neurodevelopment, suggesting that AMZ has the potential to cause developmental neurotoxicity. These findings suggest the critical importance of investigating the developmental and neurotoxic effects of AMZ on aquatic organisms. Future studies are required to evaluate the long-term effects of AMZ exposure and its broader ecological impacts on non-target species within the environment.

## Figures and Tables

**Table 1:** Primers list for real-time PCR.

Gene Name	Sequence of the Primer (5′–3′)		Accession Number
	Forward	Reverse	
*Beta-actin*	ACAGGGAAAAGATGACACAGATCA	CAGCCTGGATGGCAACGTA	NM_181601.5
*α1-tubulin*	AATCACCAATGCTTGCTTCGAGCC	TTCACGTCTTTGGGTACCACGTCA	NM_194388
*Myelin basic Protein (mbp)*	AATCAGCAGGTTCTTCGGAGGAGA	AAGAAATGCACGACAGGGTTGACG	AY860977
*Synapsin IIa (syn2a)*	GTGACCATGCCAGCATTTC	TGGTTCTCCACTTTCACCTT	NM_001002597
*Sonic hedgehog a (shha)*	GCAAGATAACGCGCAATTCGGAGA	TGCATCTCTGTGTCATGAGCCTGT	DRU30711
*gap43*	TGCTGCATCAGAAGAACTAA	CCTCCGGTTTGATTCCATC	NM_131341
*ache*	CATACGCACAATACGCTGCC	TACACAGCACCATGCGAGTT	NM_131846
*chrna7*	CCGGCAACATCTGACTCTGT	CAGTTCAACAGCACCACACG	NM_201219
*chata*	ACCGATGGTACGACAAACCC	AGAGTGTTCACAGACGACGC	NM_001130719
*hact*	CTCTCGAACCCGGCTGTATC	TATCTTCCCAAGCCATGCGG	XM_021473914
*vacht*	TACTGTATGAGTTCGCGGGC	AAGGGCTTGAGCACAGTCAG	NM_001077550
*manf*	AGATGGAGAGTGTGAAGTCTGTGTG	CAATTGAGTCGCTGTCAAACTTG	NM_001076629
*bdnf*	ATAGTAACGAACAGGATGG	GCTCAGTCATGGGAGTCC	NM_131595
*nr4a2b*	AGGCTAGAGGATCTCCGTCC	GCACCGTGCGCTTAAAGAAT	NM_001002406
*drd2b*	GATCTCCGTTGTTTGGGTGC	GCGGGGTTGGCAATTTCAC	NM_197936
*drd4a*	TGTTCGGCATCAACAACGTC	ACATTCCGCAGTACAGGAGC	NM_001012616
*drd4b*	AGCATCTCCTGTCATCTTCGG	CAGCATGAGCATAATGGGGC	NM_001012618
*drd7*	GATCTCCGTTGTTTGGGTGC	GCGGGGTTGGCAATTTCAC	NM_001113643
*tph1*	TCTGTGAACTCTACGTGTGG	CACTGGGAGCATCAGACG	AF548566
*tph2*	ATCCATCCTTGCTCTCCAAC	TCTGTGAACTCTACGTGTGG	NM_214795
*tphr*	AGATCCCATACCACACGTAGAG	CGGTTCAGGAGTGTAAAGAGG	AB125219
*serta*	ACCACCAGAGTCCTAAATGTTCCAG	CTCTTCCTTCATCTGTGTGCCTTCC	NM_001039972
*sertb*	AACCCTAACAGCAGTCCTCA	GGCCTCACCGTCACACAATA	NM_001177459
*htrlaa*	ATGAGGATGAGCGGGATGTAG	CAATCAGCCAGGACCACG	NM_001123321
*htrlab*	CTGTGTCGCCTGCACTTTTC	TGATCTCCAAAGACTCGCCG	NM_001145766

**Table 2:** Survival, hatching, and malformations in 120 hpf zebrafish larvae after exposure to AMZ.

Amicarbazone (mg/L)	Survival Rate (%)	Hatching Rate (%)	Malformation Rate (%)	Heart Rate (Beats/30s)
0	100 ± 0.0	100 ± 0.0	0.0 ± 0.0	65 ± 2.1
100	98.3 ± 0.6	100 ± 0.0	0.0 ± 0.0	65 ± 1.7
200	98.3 ± 0.6	100 ± 0.0	0.0 ± 0.0	65 ± 2.2
300	87.7 ± 6.5	94.7 ± 5.0	56.3 ± 5.5	56 ± 3.3 ***
400	59.7 ± 3.5 **	96.7 ± 2.9	94.1 ± 2.7 **	24 ± 4.4 ***

All data are expressed as the mean ± SD from three replicates (50 larvae per group in individual experiments at 120 hpf). Significant differences from the control are indicated by ** *p* < 0.01 and *** *p* < 0.001.

## Data Availability

We have full control of all primary data and agree to allow the journal to review our data if requested.

## Supplementary Materials

The following supporting information can be downloaded at: https://www.mdpi.com/article/10.3390/toxics12110783/s1, Figure S1: LC curve of AMZ. Dashed lines indicate LC_50_ and LC_20_ values.
