# Supplementary figures and images for: Neurotoxicity Assessment of Amicarbazone Using Larval Zebrafish
Source: Toxics. 2024 Oct 28;12(11):783. doi: 10.3390/toxics12110783 (PMC11598559; doi:10.3390/toxics12110783)

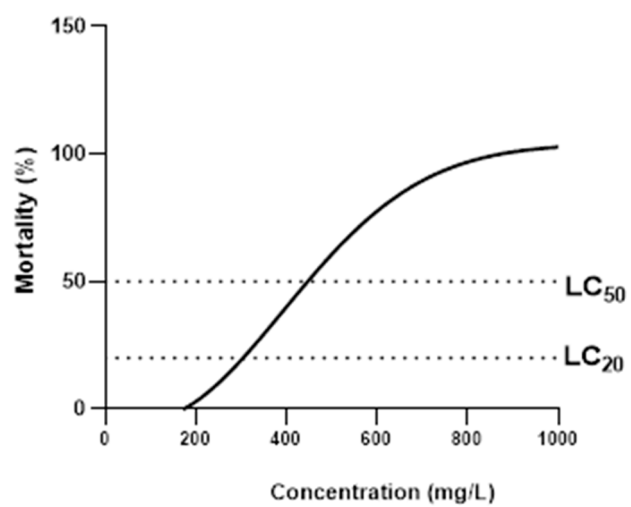

Figure S1. LC curve of AMZ. Dashed lines indicate LC<sub>50</sub> and LC<sub>20</sub> values.

Supplement: Supplementary file 1 [file toxics-12-00783-s001.zip › toxics-3224405-supplementary.pdf]
